# Beta Burst Characteristics and Coupling within the Sensorimotor Cortical‐Subthalamic Nucleus Circuit Dynamically Relate to Bradykinesia in Parkinson's Disease

**DOI:** 10.1002/mds.30163

**Published:** 2025-02-27

**Authors:** Pan Yao, Abhinav Sharma, Bahman Abdi‐Sargezeh, Tao Liu, Huiling Tan, Amelia Hahn, Philip Starr, Simon Little, Ashwini Oswal

**Affiliations:** ^1^ MRC Brain Network Dynamics Unit, University of Oxford Oxford United Kingdom; ^2^ State Key Laboratory of Transducer Technology Aerospace Information Research Institute (AIR), Chinese Academy of Sciences Beijing China; ^3^ School of Electronic, Electrical and Communication Engineering, University of Chinese Academy of Sciences (UCAS) Beijing China; ^4^ Department of Neurological Surgery Weill Institute for Neurosciences, University of California, San Francisco San Francisco CA USA

**Keywords:** beta bursts, Bradykinesia, cortico‐basal ganglia circuits, Parkinson's disease

## Abstract

**Background:**

Bursts of exaggerated subthalamic nucleus (STN) beta activity are believed to contribute to clinical impairments in Parkinson's disease (PD). No previous studies have explored burst characteristics and coupling across the sensorimotor cortical‐STN circuit and determined their relationship to dynamic measurements of bradykinesia.

**Objective:**

We sought to (1) establish the characteristics of sensorimotor cortical and STN bursts during naturalistic behaviors, (2) determine the predictability of STN bursts from motor cortical recordings, and (3) relate burst features to continuous measurements of bradykinesia using wearable sensors.

**Methods:**

We analyzed 1046 h of wirelessly streamed bilateral sensorimotor cortical and STN recordings from 5 PD patients with concurrent measurements of bradykinesia.

**Results:**

STN bursts were longer than cortical bursts and had shorter inter‐burst intervals. Long bursts (>200 ms) in both structures displayed temporal overlap (>30%), with cortical bursts tending to lead STN burst onset by 8 ms. Worsening bradykinesia was linked to increased cortical burst rates and durations, whereas STN burst properties had the opposite effect.

**Conclusion:**

Cortical beta bursts tend to precede STN beta bursts with short delays and their occurrence relates to worsening bradykinesia in naturalistic settings. © 2025 The Author(s). *Movement Disorders* published by Wiley Periodicals LLC on behalf of International Parkinson and Movement Disorder Society.

Exaggerated basal ganglia beta (13–30 Hz) frequency oscillatory synchronization is a hallmark of Parkinson's disease (PD).^1^, ^2^, ^3^ Beta activity is not continuous, often occurring in transient packets known as bursts.^4^, ^5^ Previous work has demonstrated that beta bursts of prolonged duration and amplitude correlate with bradykinesia‐rigidity components of the Unified Parkinson's Disease Rating Scale (UPDRS) score.^6^, ^7^ Furthermore, therapies such as STN deep brain stimulation (DBS) and dopaminergic medication lead to a reduction in STN beta activity, with the extent of suppression correlating with clinical improvements.^8^, ^9^ These observations have led to beta activity being used as a control signal in amplitude responsive adaptive DBS (aDBS), where STN stimulation is delivered only following the detection of beta bursts and not continuously.^10^, ^11^, ^12^, ^13^ Growing evidence suggests that aDBS may be more efficacious than continuous DBS.^13^

Although the pathophysiological significance of beta bursts is established, we still do not fully understand the mechanisms leading to burst initiation. Studies of simultaneous basal ganglia and sensorimotor cortical recordings highlight the existence of coherent beta networks in which sensorimotor cortical beta activity drives basal ganglia activity over the same frequency range.^8^, ^14^, ^15^, ^16^ This finding is consistent with canonical corticobasal ganglia circuitry^17^ and leads to the hypothesis that beta bursts originating within sensorimotor cortex could drive the onset of basal ganglia bursts, therefore reliably preceding their onset. Addressing this question is important, as it may imply predictability of the timing of basal ganglia bursts from sensorimotor cortical recordings, providing knowledge that could be used in aDBS applications or the development of less‐invasive strategies for modulating deep brain activity based on cortical stimulation.

Here we leverage an investigational DBS device capable of performing simultaneous invasive sensorimotor cortical and STN recordings in a home environment to: (1) establish the characteristics of motor cortical and STN bursts during naturalistic behaviors, (2) determine the predictability of STN bursts from cortical activity, and (3) establish the relationship between burst characteristics and continuous measurements of bradykinesia using wrist worn sensors.

## Patients and Methods

### Patient Characteristics

**FIG. 1:**
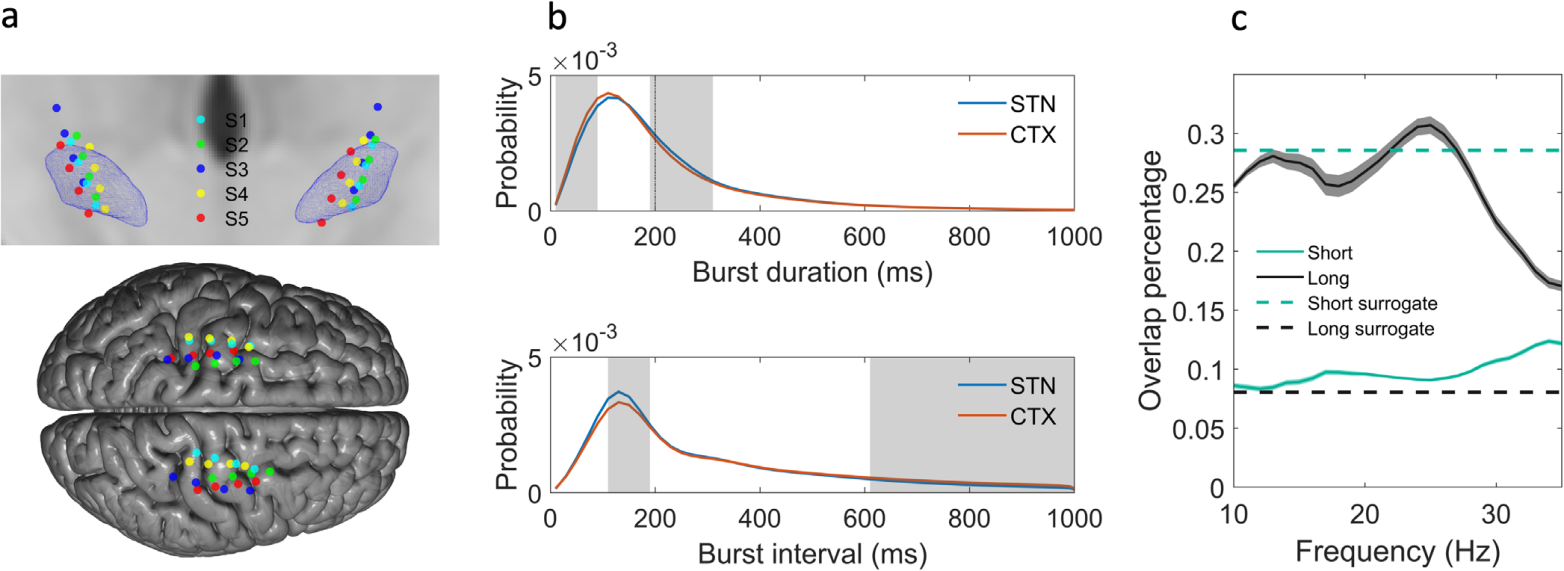
Electrode contact locations and beta burst characteristics and coupling within the motor cortical‐subthalamic nucleus (STN) circuit. (**A**) The upper image shows STN electrode contact locations for all 5 patients (S1–S5), superimposed on an STN mesh (blue) and template MRI (coronal view) in Montreal Neurological Institute (MNI) space. The lower image shows cortical contact locations, superimposed on a cortical mesh derived from the same template MRI. (**B**) Probability density functions (pdfs) for STN and motor cortical burst durations and inter‐burst intervals. The dotted black line in the burst duration pdf indicates the 200 ms cutoff that was used to differentiate long and short bursts. There was a higher proportion of short duration bursts (10–90 ms) in the cortex and a correspondingly higher proportion of longer duration bursts (190–310 ms) within the STN, as indicated by the gray‐shaded regions. Similarly, the probability of inter‐burst intervals between 110 and 190 ms was greater in the STN, whereas the probability of intervals greater than 610 ms was greater in the motor cortex. (**C**) The overlap of cortical bursts relative to STN bursts for frequencies in the range of 10–35 Hz (1 Hz resolution ±3 Hz), separately for long (>200 ms) and short (<200 ms) burst durations. For long bursts, the maximal overlap was greater than 0.3 and occurred at a frequency of 25 Hz. Mean values are shown with shaded areas indicating the standard error of the mean. The dashed lines indicate the 99th percentile of overlaps derived from surrogate data that were generated using the pdfs of observed data plotted in (a). Surrogate data overlaps were computed separately for long and short bursts. [Color figure can be viewed at wileyonlinelibrary.com]

We analyzed data from five patients with PD (see Table S1), with bilateral implants of the Summit RC + S neural interface (Medtronic). Each STN was implanted with a quadripolar Medtronic 3389 lead, whereas cortical recordings were performed using a quadripolar paddle‐type lead (10 mm intercontact spacing), such that 2–3 contacts were anterior to the central sulcus, 2–4 cm lateral from the midline. STN and cortical contact locations were confirmed by fusion (linear co‐registration) of post‐operative CT and pre‐operative 3 T MRI imaging as previously reported.^18^, ^19^ Group visualization was then performed by normalizing all electrode contact locations into Montreal Neurological Institute space using the Lead‐DBS toolbox^20^ (see Fig. 1A).

Data from two bipolar STN channels (formed from contacts 1–3 and 2–4; with contacts labeled anatomically in order from inferior to superior) and two bipolar cortical channels (formed from contacts 1–3 and 2–4; with contacts labeled anatomically in order from anterior to posterior) were streamed from each hemisphere (sampling rate of 250 Hz; see Supporting Information Methods for further recording details) to a Microsoft Windows tablet.^18^, ^19^ In all cases at least one STN contact was located within the sensorimotor STN, and one cortical contact was situated over the motor cortex (see Fig. 1A). Recordings were completed between 2 and 4 weeks after implantation, during activities of daily living, while patients were on their usual dopaminergic medication, before the initiation of DBS. During recordings patients wore Global Kinetics Pty Ltd. Personal KinetiGraph® (PKG®) monitors on both wrists, as previously reported,^18^ which provided continuous measurements of bradykinesia, tremor, and dyskinesia in 2‐minute intervals. This study was approved by the University of California, San Francisco, institutional review board (IRB).

### Defining Burst Characteristics and the Overlap of Cortico‐STN Bursts

Data from each STN and cortical channel were bandpass filtered (±3 Hz using a fourth‐order zero‐phase Butterworth filter) around the beta peak frequency for cortico‐STN coherence for each hemisphere (see Table S1 for further details). This allowed us to consider bursts at the same frequency that were synchronized across sites. Beta burst timings were defined separately for each channel as time points where the beta amplitude envelope exceeded its 75th percentile (see Fig. S1 for example data). Although the 75th percentile is a standard threshold used in the beta burst literature,^4^, ^5^, ^21^, ^22^, ^23^ we also explored the effects of using thresholds of 70% and 80%. Burst duration and inter‐burst interval probability density functions were then computed using 20 ms spaced bins for values ranging from 0 to 1000 ms. A general linear model (GLM; using spm_ancova.m,^24^ with covariates added to account for between and within [hemispheric] subject differences) enabled comparison of cortical and STN burst durations and inter‐burst intervals at each time bin, with cluster‐based permutation testing being used to correct for multiple comparisons (cluster forming and cluster extent thresholds set to *P* < 0.01).

Bursts were also separately defined for each integer frequency (±3 Hz window) ranging from 10 to 35 Hz. For each frequency and for each of the four combinations of STN and cortical channels from each hemisphere, we computed the overlap of STN and cortical bursts. This was defined as the total number of time points during which there was a burst in both sites, divided by the number of time points during which an STN burst occurred. Overlap proportions were computed separately for short (<200 ms) and long (>200 ms) duration bursts, as only the latter are believed to adopt a pathological role.^4^, ^5^, ^21^

### Generation of Surrogate Data

Surrogate data allowed us to determine whether observed cortico‐STN burst overlap profiles were greater than above chance for both long and short duration bursts. Based on the probability of each 20 ms wide burst duration bin (Fig. 1A) a fixed number of bursts for each bin duration was generated and used to create binarized burst timing data that preserved the original burst characteristics for both sites. For each instantiation of surrogate data for both STN and cortical sites, the ordering of bursts was randomized, and inter‐burst intervals were drawn from corresponding density functions (Fig. 1A). About 1000 instantiations of surrogate data were used to compute null distributions for statistical testing.

### Directionality of Cortico‐STN Burst Coupling

**FIG. 2:**
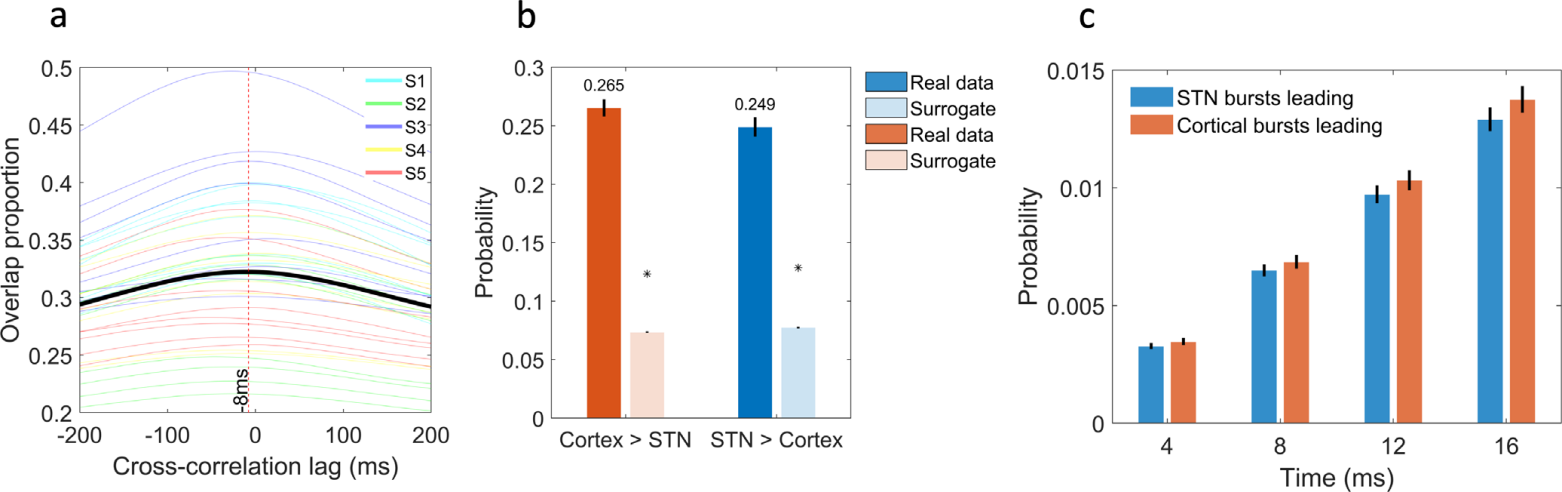
Cortical bursts at the peak overlap frequency tend to precede subthalamic nucleus (STN) bursts. (**A**) The cross‐correlation function of the overlap of long cortical bursts relative to STN bursts at a frequency of 25 ± 3 Hz. Data are shown for individual combinations of STN and cortical channels (four combinations from two bipolar STN and two bipolar cortical channels) for each hemisphere of each subject (the colors S1–S5 indicate data from each of the 5 patients), with the black line indicating the mean. The maximal overlap is achieved when STN data are shifted—8 ms relative to cortical data—indicative of a cortico‐STN burst conduction time of 8 ms (see dashed red line). (**B**) The proportion of long STN bursts (at a center frequency of 25 Hz) that were preceded by a cortical burst and vice versa for both the real data and surrogate data. The bars indicate mean values, whereas black lines indicate standard errors. The stars indicate the 99th percentile derived from surrogate data distributions. The onset of 26.5% of all STN bursts was preceded by the onset of a cortical burst, while the corresponding figure for STN bursts preceding cortical bursts was 24.9%. Both values were above chance (*P* < 0.01). **(C)** The proportion of STN bursts (at a center frequency of 25 Hz) whose onset was preceded by the onset of a cortical burst (and vice versa) within different timeframes (4, 8, 12, and 16 ms) is shown. Bars indicate mean values, whereas the black lines represent standard errors. A two‐factor analysis of variance (ANOVA) revealed that STN bursts are more likely to be preceded by cortical bursts than vice versa (T_303_ = 2.47, *P* = 0.007; see Results). [Color figure can be viewed at wileyonlinelibrary.com]

To determine whether long cortical bursts tend to precede long STN bursts, we computed the burst overlap proportion at different time lags for STN timeseries shifts relative to cortical activity. This was performed for the frequency displaying the largest cortico‐STN burst overlap. A one‐sample *t* test was then used to test for statistical significance. Additionally, for both sites, we computed the proportion of long bursts in one site whose onset was accompanied by and also preceded by a burst in the other site. This was performed separately for both the real and surrogate datasets. Finally, in view of the overlap being maximal at a lag of −8 ms (see Fig. 2A), we computed the proportion of long bursts in one site that were preceded by the onset of a burst in the other site within time windows of 4, 8, 12, and 16 ms.

### Analysis of PKG® and Neural Data

PKG® and neural data were synchronized using recording timestamps as previously described.^18^, ^19^ Crucially, behavioral analysis used contralateral wrist data—for example, neural data from the left hemisphere were paired with right wrist data and vice versa. Bradykinesia scores were first transformed from negative to positive, prior to data corresponding to periods of sleep being excluded (using immobility as a surrogate marker; >2 minutes of immobility with a bradykinesia score > 80).^18^ The following beta burst properties—computed as before, but derived from 2‐minute long windows preceding each bradykinesia readout—were used to predict bradykinesia in a GLM: (1) STN and cortical beta burst rates (bursts per minute), (2) STN and cortical mean burst lifetimes (defined as the mean duration of bursts within each 2‐minute window), and (3) the cortico‐STN beta burst overlap proportion. Burst properties were normalized to a value between 0 and 1, and covariates were included to account for subject and hemisphere specific effects. Normalization is a standard practice for data variables with large differences in scale that can facilitate comparison of regression coefficients.^18^, ^25^

## Results

### Sensorimotor Cortical and STN Beta Burst Characteristics

Probability density functions for cortical and STN burst durations and inter‐burst intervals are displayed in Figure 1A. Gray regions indicate the presence of significant differences between the STN and cortex. We observed a greater proportion of short duration (10–90 ms) bursts within the sensorimotor cortex and a correspondingly higher proportion of long duration (190–310 ms) bursts within the STN. Furthermore, inter‐burst intervals tended to be shorter within the STN than within the sensorimotor cortex. In Figure 1, the 75th percentile was used as the amplitude threshold for defining beta bursts. Results using thresholds of 70% and 80% are presented in Figure S2. It can be seen that the differences in burst characteristics between the STN and cortex persist at different thresholds.

### Cortico‐STN Burst Overlap and Estimation of Cortico‐STN Transmission Delays

Long bursts (>200 ms) displayed a greater overlap between the sensorimotor cortex and STN than short bursts (<200 ms), such that the maximal overlap for long bursts was greater than 30% at the high beta frequency of 25 Hz (Fig. 1B). Overlap proportions were also derived for surrogate data, with Figure 1B showing the 99th percentile of the surrogate distribution for long and short burst overlaps. Only long bursts displayed above chance coupling at all frequencies between 10 and 35 Hz (significant when corrected for multiple comparisons using cluster‐based permutation testing with cluster forming and cluster extent thresholds of *P* < 0.01). Cortico‐STN burst overlap plots, computed using burst definition thresholds of the 70th and 80th percentiles, also exhibited similar profiles (see Fig. S2).

Cross‐correlation of cortical and STN burst time courses at the peak overlap frequency of 25 Hz revealed a maximal overlap at a mean lag of −8 ms. We confirmed that this mean value was significantly less than 0, using a one sample *t* test (T_30_ = 8.16, *P* < 0.01). This result suggests that cortical bursts tend to precede STN bursts by approximately 8 ms (Fig. 2A). A similar lag of −12 ms was also observed for the two different burst definition thresholds (see Fig. S2).

### Cortical Bursts Precede STN Bursts

Figure 2B highlights that at the center frequency of 25 Hz, 26.5% of long STN bursts were preceded by the onset of a sensorimotor cortical burst. Interestingly, we also observed a reciprocal relationship, such that ~25% of long cortical bursts were preceded by the onset of an STN burst. Both of these percentages were above chance values (*P* < 0.01 indicated by the stars in Fig. 2B) derived from surrogate data. This result highlights that long bursts in the STN can be preceded by a burst in the cortex, but also vice versa.

Next, we tested whether cortical bursts were more likely to precede long STN bursts than STN bursts preceding long cortical bursts. Given that cross‐correlation lags resulting in maximal overlap tended to be in the range of 4–16 ms (see Fig. 2A), we explored whether bursts in one site were preceded by a burst within the other site for values within this time frame. The results, shown in Figure 2C, highlight that cortical bursts are more likely to precede STN bursts than vice versa. This was confirmed using a two‐factor analysis of variance (ANOVA), with factors leading site (levels: STN leading vs. cortex leading) and precession timeframe (levels: 4 ms vs. 8 ms vs. 12 ms vs. 16 ms). There was a main effect of leading site (T_303_ = 2.47, *P* = 0.007) such that the cortex was more likely to lead than the STN.

### Beta Burst Characteristics Dynamically Corelate with Bradykinesia

**FIG. 3:**
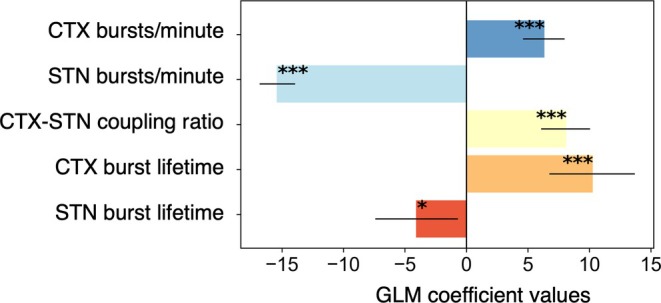
Beta burst properties correlate with dynamic measures of bradykinesia. A general linear model (GLM) was applied to explore the relationship between burst properties in each hemisphere and contralateral bradykinesia scores, measured in 2‐minute windows using Personal KinetiGraph® (PKG). To simplify interpretation, the raw PKG bradykinesia scores were multiplied by −1, converting them to a positive scale. The *x*‐axis displays the beta coefficients derived from the GLM, with each bar representing the coefficient value for the burst property listed on the *y*‐axis. Positive coefficients indicate an association with higher bradykinesia scores, whereas negative coefficients suggest an association with lower scores. Error bars denote the 95% confidence intervals for the coefficients. Statistically significant associations are highlighted with *** (*P* < 0.001) and * (*P* < 0.05). Cortical burst properties were positively associated with bradykinesia, whereas STN burst properties showed a negative association. Additionally, increased cortico‐subthalamic nucleus (STN) burst coupling was linked to worsening bradykinesia. [Color figure can be viewed at wileyonlinelibrary.com]

Figure 1B shows group spectra for data divided into periods of high bradykinesia (>70th percentile of bradykinesia score), low bradykinesia (<70th percentile of bradykinesia score), and sleep, based on PKG. Beta activity is subtly reduced during states of low bradykinesia and sleep. GLM analysis revealed that cortical burst properties (burst rates and lifetimes) were positively associated with worsening bradykinesia (Fig. 3). Additionally, increased coupling of cortical and STN bursts was detrimental to bradykinesia (T_22300_ = 5.76, *P* < 0.001 for cortical burst lifetime; T_22300_ = 7.30, *P* < 0.001 for cortical burst rate; T_22300_ = 7.96, *P* < 0.001 for cortico‐STN burst coupling). Interestingly for the STN, the opposite effect was observed, such that both increased burst rates and increased burst lifetimes were associated with ameliorations of bradykinesia (T_22300_ = −2.36, *P* = 0.018 for STN burst lifetime; T_22300_ = −20.83, *P* < 0.001 for STN burst rate)—see Discussion for further comment. The coefficient of determination (R^2^) of the full model was 0.15.

## Discussion

We leveraged prolonged invasive recordings of sensorimotor cortical and STN activity during daily living, to interrogate the characteristics of cortical and STN beta bursts, and to relate these features to dynamic recordings of bradykinesia. To our knowledge this is the first study to reveal differences in the properties of motor cortical and STN beta bursts in PD–with STN bursts tending to be longer and having shorter inter‐burst intervals. Additionally, our findings imply significant cross‐site coupling of long bursts, with an overlap percentage of >30% at high beta (25 Hz) frequencies. Notably, this overlap value is markedly greater than that reported in previous studies, where cortical activity has been recorded (with lower signal‐to‐noise ratio) using EEG.^22^ Nevertheless, our results also reveal that STN bursts most commonly occur in the absence of a concurrent cortical burst, a finding that may suggest a role for subcortical pathways in STN burst propagation (eg, striatal pathways or the reciprocal STN‐GPe loop^26^, ^27^, ^28^).

Cross‐correlation analyses revealed that long cortical bursts tend to precede the onset of long STN bursts, with an estimated conduction delay of 8 ms. Although this value is consistent with reports of monosynaptic hyperdirect pathway latencies between the sensorimotor cortex and the STN,^29^ we note that delays estimated from cross‐correlation (and similar) analyses of biological networks should be interpreted with a degree of caution.^30^ Interestingly, we also observed that STN bursts were more likely than chance to precede cortical bursts. This is perhaps unsurprising given the presence of reciprocal anatomical connectivity between the STN and sensorimotor cortex via thalamo‐cortical circuits.^31^

Importantly, our results also reveal for the first time that beta burst properties correlate dynamically with bradykinesia. The prolongation of cortical burst duration and rate was associated with worsening bradykinesia, as was increased cortico‐STN burst coupling. Conversely, however, increased STN burst rates and durations were associated with ameliorations of bradykinesia. One possible explanation for this finding is that our data were recorded in naturalistic settings while patients took their usual dopaminergic medication. Previous work has suggested that dopaminergic medication reduces the occurrence of antikinetic long duration bursts within the STN, while simultaneously enhancing the occurrence of short duration bursts that may have a physiological or prokinetic role.^4^, ^5^ The enhancement of prokinetic short bursts may undlie the direction of our observed association between STN burst features and bradykinesia.

Overall, our results imply that long beta bursts are coupled across the sensorimotor cortical‐STN circuit, with cortical bursts tending to precede the onset of STN bursts. Furthermore, the prolongation of cortical burst rates, durations, and cortico‐STN burst coupling is dynamically related to worsening bradykinesia. These findings also lead us to speculate that suppressing cortical bursts, via non‐invasive or minimally invasive approaches, could lead to improvements in motor symptoms in naturalistic states.

## Author Roles

(1) Research project: A. Conception, B. Organization, C. Execution; (2) Statistical analysis: A. Design, B. Execution, C. Review and critique; (3) Manuscript preparation: A. Writing of the first draft, B. Review and critique.

P.Y.: 1ABC, 2ABC, 3AB

A.S.:1BC, 2ABC, 3B

B.A.:1BC, 2C, 3B

T.L.:1C, 2C, 3B

H.T.:1C, 2C, 3B

A.H.:1C, 2C, 3B

P.S.: 1ABC, 2C, 3B

S.L.:1ABC, 2C, 3B

A.O.: 1ABC, 2ABC, 3B

## Full financial disclosures for the previous 12 months

P.S. is a consultant for Neuralink and InBrain Neuroelectronics. S.L. is a consultant for Iota Biosciences.

## Supporting information


**Supplementary Figure S1.** Exemplar data and group spectra of subthalamic nucleus (STN) and cortical channels in states of high and low bradykinesia. (**A**) Exemplar cortical and STN data are presented from the left hemisphere of patient 3 after bandpass filtering ±3 Hz around 26 Hz (the beta peak frequency for cortico‐STN coherence; see also Table S1). The envelope of the filtered data (blue) is plotted in red, whereas the threshold amplitude for burst definition (75th percentile of the amplitude envelope) is indicated by the horizontal yellow lines. In the upper plot, the black horizontal lines indicate time periods during which an STN beta burst occurred, whereas the dark red lines indicate time periods during which STN bursting and cortical bursting overlapped. (**B**) Mean spectra computed across all STN and cortical bipolar channels from all subjects. Data have been divided into periods of high and low bradykinesia as well sleep based on Personal KinetiGraph® (PKG)‐derived bradykinesia scores. High bradykinesia data segments include those where the bradykinesia score was above the 70th percentile. The remaining data segments are considered to correspond to a low bradykinesia state. Sleep was classified on the basis of patient immobility, defined as a PKG bradykinesia score of greater than 80. Beta activity in both the cortex and the STN is higher during high bradykinesia states compared to low bradykinesia states.

**Supplementary Figure S2.** Beta burst characteristics and coupling determined using two different beta amplitude envelope thresholds (70th and 80th percentiles) for burst definition. (**A**) Probability density functions (pdfs) for subthalamic nucleus (STN) and motor cortical burst durations. The dotted black line in the burst duration pdf indicates the 200 ms cutoff that was used to differentiate long and short bursts. There was a higher proportion of short duration bursts (10–130 ms for the 70% threshold and 10–50 ms for the 80% threshold) in the cortex and a correspondingly higher proportion of longer duration bursts (210–490 ms for the 70% threshold and 170–230 ms for the 80% threshold) within the STN, as indicated by the gray shaded regions. (**B**) PDFS of inter‐burst intervals for the two different thresholds. Inter‐burst intervals tended to be shorter in the STN than in the cortex. For the 70% threshold the probability of intervals between 70 and 170 ms was greater in the STN and the probability of intervals longer than 370 ms was greater in the cortex. Similarly for the 80% threshold, the probability of intervals between 150–190 and 250–330 ms was greater in the STN, whereas the probability of intervals greater than 710 ms was highest in cortex. (**C**) The overlap of cortical bursts relative to STN bursts for frequencies in the range of 10–35 Hz (1 Hz resolution ±3 Hz), separately for long (>200 ms) and short (<200 ms) burst durations and for the two different burst definition thresholds. For long bursts, the maximal overlap was greater than 0.25 and occurred at a frequency of 25 Hz. Increasing the threshold reduced the overlap proportion but did not change the profile of the peaks for long duration bursts. Mean values are shown with shaded regions indicating the standard error of the mean. (**D**) The cross‐correlation function of the overlap of long cortical bursts relative to STN bursts at a frequency of 25 ± 3 Hz. Mean data across subjects (four combinations from two bipolar STN and two bipolar cortical channels for each hemisphere of each subject) are shown for the two different burst definition thresholds. The maximal overlap is achieved when STN data are shifted—12 ms relative to cortical data—indicative of a cortico‐STN burst conduction time of 12 ms (see the dashed red line).

**Data S1**. supporting Information.

## Data Availability

The data that support the findings of this study are available on request from the corresponding author. The data are not publicly available due to privacy or ethical restrictions.
