# Supplementary figures and images for: Beta Burst Characteristics and Coupling within the Sensorimotor Cortical‐Subthalamic Nucleus Circuit Dynamically Relate to Bradykinesia in Parkinson's Disease
Source: Mov Disord. 2025 Feb 27;40(5):962–8. doi: 10.1002/mds.30163 (PMC12089894; doi:10.1002/mds.30163)

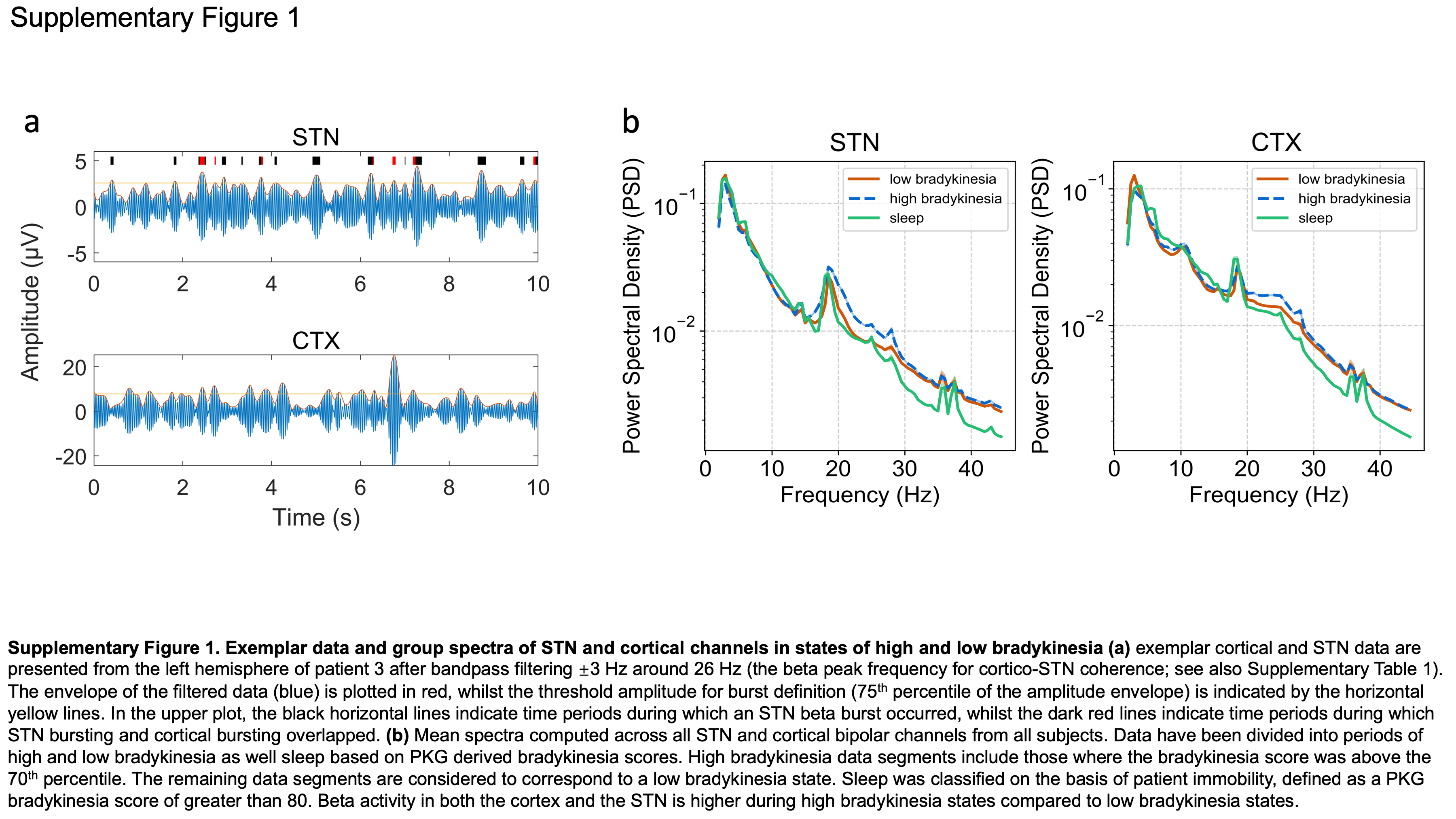

Supplement: Supplementary file 1 — Supplementary Figure S1. Exemplar data and group spectra of subthalamic nucleus (STN) and cortical channels in states of high and low bradykinesia. (A) Exemplar cortical and STN data are presented from the left hemisphere of patient 3 after bandpass filtering ±3 Hz around 26 Hz (the beta peak frequency for cortico‐STN coherence; see also Table S1). The envelope of the filtered data (blue) is plotted in red, whereas the threshold amplitude for burst definition (75th percentile of the amplitude envelope) is indicated by the horizontal yellow lines. In the upper plot, the black horizontal lines indicate time periods during which an STN beta burst occurred, whereas the dark red lines indicate time periods during which STN bursting and cortical bursting overlapped. (B) Mean spectra computed across all STN and cortical bipolar channels from all subjects. Data have been divided into periods of high and low bradykinesia as well sleep based on Personal KinetiGraph® (PKG)‐derived bradykinesia scores. High bradykinesia data segments include those where the bradykinesia score was above the 70th percentile. The remaining data segments are considered to correspond to a low bradykinesia state. Sleep was classified on the basis of patient immobility, defined as a PKG bradykinesia score of greater than 80. Beta activity in both the cortex and the STN is higher during high bradykinesia states compared to low bradykinesia states. [file MDS-40-962-s003.tiff]

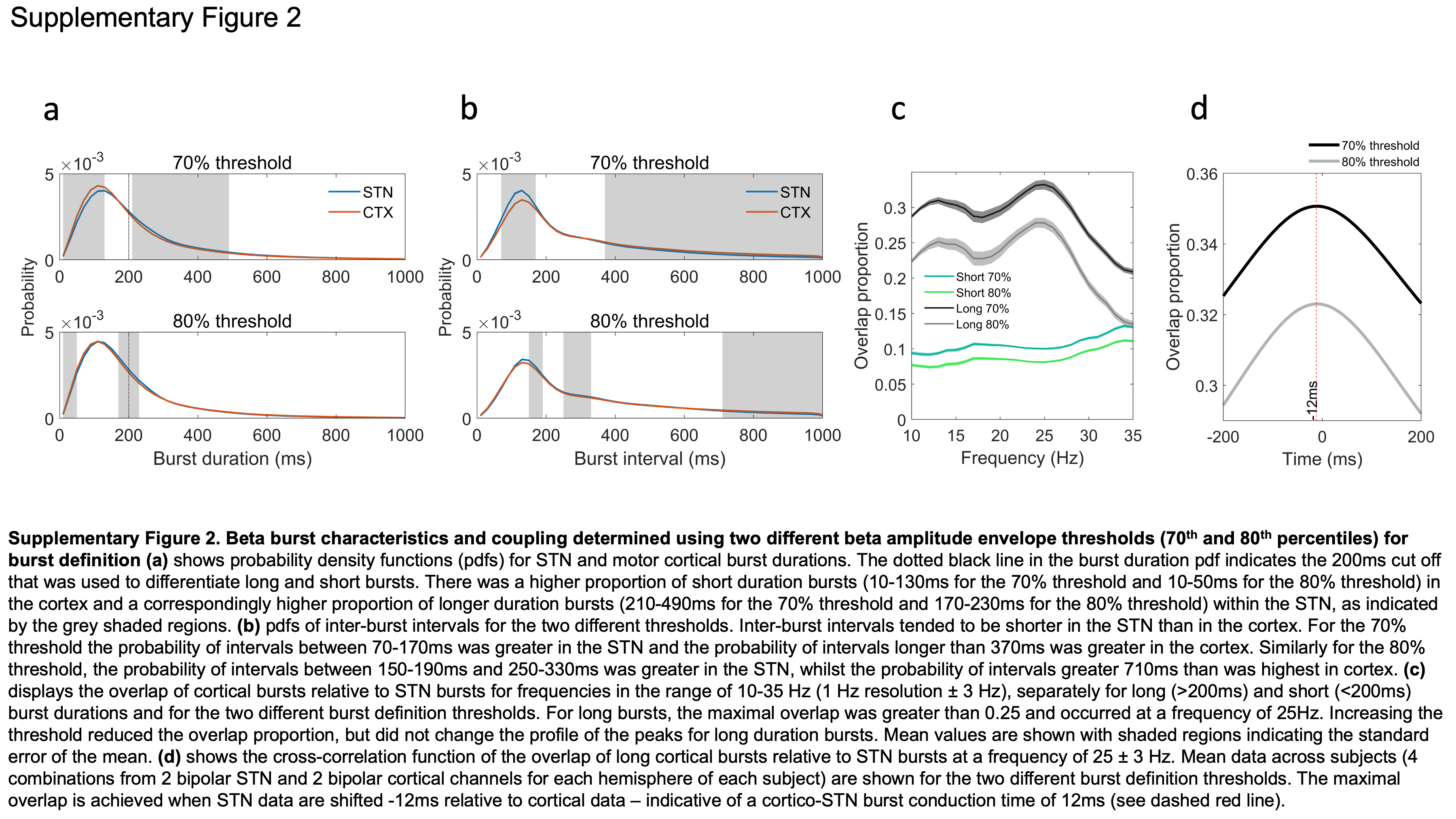

Supplement: Supplementary file 2 — Supplementary Figure S2. Beta burst characteristics and coupling determined using two different beta amplitude envelope thresholds (70th and 80th percentiles) for burst definition. (A) Probability density functions (pdfs) for subthalamic nucleus (STN) and motor cortical burst durations. The dotted black line in the burst duration pdf indicates the 200 ms cutoff that was used to differentiate long and short bursts. There was a higher proportion of short duration bursts (10–130 ms for the 70% threshold and 10–50 ms for the 80% threshold) in the cortex and a correspondingly higher proportion of longer duration bursts (210–490 ms for the 70% threshold and 170–230 ms for the 80% threshold) within the STN, as indicated by the gray shaded regions. (B) PDFS of inter‐burst intervals for the two different thresholds. Inter‐burst intervals tended to be shorter in the STN than in the cortex. For the 70% threshold the probability of intervals between 70 and 170 ms was greater in the STN and the probability of intervals longer than 370 ms was greater in the cortex. Similarly for the 80% threshold, the probability of intervals between 150–190 and 250–330 ms was greater in the STN, whereas the probability of intervals greater than 710 ms was highest in cortex. (C) The overlap of cortical bursts relative to STN bursts for frequencies in the range of 10–35 Hz (1 Hz resolution ±3 Hz), separately for long (>200 ms) and short (<200 ms) burst durations and for the two different burst definition thresholds. For long bursts, the maximal overlap was greater than 0.25 and occurred at a frequency of 25 Hz. Increasing the threshold reduced the overlap proportion but did not change the profile of the peaks for long duration bursts. Mean values are shown with shaded regions indicating the standard error of the mean. (D) The cross‐correlation function of the overlap of long cortical bursts relative to STN bursts at a frequency of 25 ± 3 Hz. Mean data across subjects (four combinations from two bi [file MDS-40-962-s001.tiff]
