# Supplementary material for: Beta Burst Characteristics and Coupling within the Sensorimotor Cortical‐Subthalamic Nucleus Circuit Dynamically Relate to Bradykinesia in Parkinson's Disease
Source: Mov Disord. 2025 Feb 27;40(5):962–8. doi: 10.1002/mds.30163 (PMC12089894; doi:10.1002/mds.30163)
Supplement: Supplementary file 3 — Data S1. supporting Information. [file MDS-40-962-s002.docx]

**Supplementary Materials**

**Supplementary Methods**

**Details of cortical and STN recordings**

All 5 patients underwent bilateral implantation of quadripolar deep brain stimulation leads into the subthalamic nucleus (Medtronic model 3389, 1.5 mm contact length and 2.0 mm intercontact spacing), bilateral placement of paddle-type quadripolar cortical paddles into the subdural space over motor cortex (Medtronic model 0913025, 4 mm contact diameter and 10 mm intercontact spacing), and bilateral placement of investigational sensing implantable pulse generators (IPGs) in a pocket over the pectoralis muscle (Medtronic Summit RC+S model B35300R). The IPG and leads were connected by 60 cm lead extenders (Medtronic model 37087), two on each side.

The Summit RC+S is a rechargeable bidirectional neural interface that offers the researcher a great degree of flexibility through access to the device’s application programming interface (API)^1^. For all research functions including sensing, investigators controlled the device by writing software in C# within the device API, accessed using a “research development kit” (RDK, Medtronic model 4NR013) provided by the manufacturer. One application was a patient-facing graphical user interface (GUI) that allowed the patient to control streaming in a home environment (this is made available at <https://openmind-consortium.github.io>.).

Patients initiated home recordings using the patient-facing GUI on a Microsoft Surface Go computer with broadband cellular service. A lightweight telemetry bridge was carried by patients to transmit data received from the IPG in the MICS-band (Medical Implant Communication Service) short range radio to an encrypted Bluetooth signal. The Bluetooth signal was then received by the computer, which automatically connected when patients were in range (approximately 12 meters)^2^. Streamed data contained no personal health information. Data were encrypted and uploaded to a secure cloud environment operated by UCSF. Patients collected data in 1–2-week recording “sprints” in which they were instructed to carry the computer with them and stream continuously if possible. The RC+S device employs a User Datagram Protocol (UDP) to transmit data to an external tablet in packets (average duration 50 ms). Occasionally (1-5%), packets are dropped or lost in transmission e.g., when the patient walks out of the 12m range of the recording tablet. As we did not interpolate dropped packets, but removed them, recordings were divided into multiple sessions. Freely available software, as previously described, was used to account for dropped packets (https://github.com/openmind-consortium/Analysis-rcs-data) ^2,3^.

The Summit RC+S device has configurable device filters^1^ that must be selected. All filters are applied after digitization, and low pass filters are applied twice – before and after amplification (gain of 250). In the absence of therapeutic stimulation, we used a high pass filter of 0.85 Hz and low pass filter of 450 Hz before amplification and 1700 Hz after amplification. Of note the device has a noise floor of approximately 100nV/√Hz, with a minimum detectable signal estimate of <300nVrms ^1^.

**References**

1. Stanslaski, S. *et al.* A Chronically Implantable Neural Coprocessor for Investigating the Treatment of Neurological Disorders. *IEEE Trans Biomed Circuits Syst* **12**, 1230–1245 (2018).

2. Gilron, R. *et al.* Long-term wireless streaming of neural recordings for circuit discovery and adaptive stimulation in patients with Parkinson’s disease HHS Public Access Author manuscript. *Nat Biotechnol* **39**, 1078–1085 (2021).

3. Sellers, K. K. *et al.* Analysis-rcs-data: Open-Source Toolbox for the Ingestion, Time-Alignment, and Visualization of Sense and Stimulation Data From the Medtronic Summit RC+S System. *Front Hum Neurosci* **15**, 714256 (2021).

**Supplementary Table 1**

| **Patient** | **Age and gender** | **Disease duration (years)** | | **﻿Preoperative medication (mg)** | **UPDRS III off medication** | **% Change in UPDRS III when on** | **MOCA** | **Data duration with concurrent contralateral PKG (hours)** | **Beta peak frequency for cortico-STN coherence** |
| --- | --- | --- | --- | --- | --- | --- | --- | --- | --- |
| 1 02 | 54 M | | 7 | LDE 1425 | 49 | 90% | 26 | L: 123.7  R: 192.9 | L: 22 Hz  R: 26 Hz |
| 2 05 | 63 M | | 19 | LDE 955 | 45 | 51% | 30 | L: 27.7  R: 24.0 | L: 30 Hz  R: 30 Hz |
| 3 06 | 28 F | | 12 | LDE 1550 | 61 | 73% | 27 | L: 80.5  R: 162.7 | L: 26 Hz  R:28 Hz |
| 4 07 | 40 M | | 4 | LDE 1314 | 41 | 65% | 30 | L: 192.7  R: 175.6 | L: 25 Hz  R: 25 Hz |
| 5 08 | 58 M | | 12 | LDE 2100 | 44 | 75% | 27 | L: 40.4  R: 25.7 | L: 24 Hz  R: 24 Hz |

**Clinical characteristics of patients.** LDE = levodopa dose equivalent. The total pre-operative UPDRS part III score is presented in the off-medication state. The beta peak frequency for cortico-STN coherence was calculated as the peak value within the frequency range (13-30Hz). Coherence was calculated using a multitaper approach (implemented in the FieldTrip toolbox; <https://www.fieldtriptoolbox.org/>), with a frequency resolution of 1Hz and a taper smoothing frequency of 2 Hz.
